# Effect of iron-fortified jamun leather on the Asunra-induced anemia in Sprague Dawley rats

**DOI:** 10.3389/fnut.2023.1195981

**Published:** 2023-06-13

**Authors:** Nosheen Naz, Moazzam Rafiq Khan, Muhammad Asim Shabbir, Muhammad Naeem Faisal

**Affiliations:** ^1^National Institute of Food Science and Technology, University of Agriculture, Faisalabad, Pakistan; ^2^Institute of Pharmacy, Physiology, and Pharmacology, University of Agriculture, Faisalabad, Pakistan

**Keywords:** fortification, jamun leather, biochemical analysis, histopathology, iron deficiency

## Abstract

**Introduction:**

Micronutrients such as minerals and vitamins are required in a minute quantity but play a pivotal role in the functioning of the body. Therefore, deficiency in one of them can lead to lethal health conditions. Iron deficiency anaemia is one of the most common micronutrient deficiencies across the world and is affecting women and children.

**Methods:**

The present study aimed to investigate the anti-anaemic effect of fortified jamun leather on anaemia biomarkers and haematology in anaemic female Sprague Dawley rats. A total of 40 Sprague Dawley rats were used in 4 groups. Iron deficiency anaemia was induced by oral administration of the Asunra drug. The treatments were fed at two dosage levels i.e., 40 and 60% iron-fortified leather. All animals were treated for 60 days and the parameters including biochemical, and histopathology of the kidney and liver were examined.

**Results:**

The experiment's findings showed that the group fed with iron-fortified leather (G_3_) succeeded significantly (*P* < 0.05) in restoring the serum iron (98.68 ± 2.88 μg/dL), haemoglobin (12.41 ± 0.32 g/dL), ferritin (24.54 ± 1.98 ng/mL) and haematocrit levels (39.30 ± 1.66%) at the end of the 60 days period. Additionally, the treated group's mean values for transferrin and total iron binding capacity were lower than those of the anaemic rats, indicating an improvement in iron levels. The microscopic analysis revealed that treatments had no toxic effects on the kidney and liver tissues, except in the diseased group, which had necrosis and irregular cell structure.

**Conclusion:**

Conclusively, iron-fortified jamun leather helped improve iron deficiency biomarkers and imparted a non-toxic effect on tissues in rats.

## Graphical Abstract

Effect of iron-fortified jamun leather on Asunra induced anaemia in Sprague Dawley rats. 
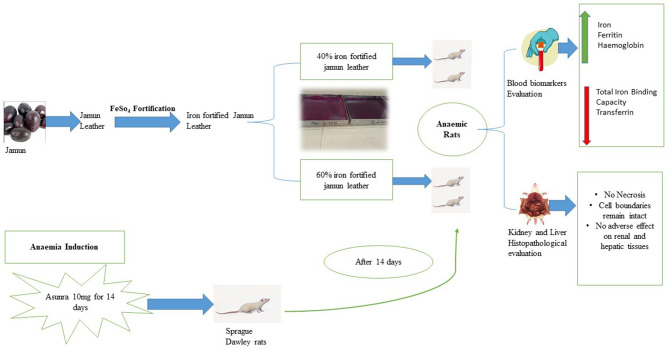

## 1. Introduction

Micronutrient deficiencies in humans have become increasingly common, especially among the population of poor socioeconomic settings (1). According to WHO, iron deficiency anemia is the most common nutritional deficiency globally (2). Although iron deficiency anemia (IDA) is more prevalent in children and women, adult men are also likely to be susceptible depending on their health and socioeconomic status (3). The main causes of anemia are genetic issues or nutrient deficiencies. Iron deficiency is the main reason for anemia and participates in 50% of gross anemic cases globally (4). Factors such as menstruation in women, gastrointestinal issues, decreased intake of dietary iron, and low absorption of iron in the body are responsible for iron deficiency anemia (5). Iron is an essential micronutrient and is regulated by factors such as dietary levels, absorption, and the recycling of iron (6). Tannins, oxalates, polyphenols, and phytates hinder the absorption of elemental iron in the body. On the other hand, gastric acid, citrate, and ascorbic acid are the facilitators to enhance absorption in the body (7). Iron is required for cellular functioning, enzymatic regulation, oxygen transport, mitochondrial energy generation, and DNA synthesis (8).

The mitigation of deficiencies is crucial and is globally addressed through different strategies such as food fortification, dietary diversification, and food supplement (9). Among all the methods, fortification is one of the most effective methods to provide the micronutrient to the compromised population (10). Food fortification is the process of enhancing food's nutritional value by adding micronutrients, which helps to alleviate nutritional deficiencies caused due to poor diet. Moreover, it is a practical, long term, and more feasible approach to improving the micronutrient status of the malnourished population (11). Food fortification has significantly increased the availability of essential nutrients such as iron, folate, vitamin A, and iodine. Regular intake of fortified food products helps in the consistent supply of nutrients to the body (12). A variety of iron fortificants has been used in the process of fortification, ferrous sulfate is the commonly used low-cost fortificant. Several studies have reported positive clinical effects of ferrous sulfate on blood biomarkers and the body (4).

Jamun (*Syzygium cumini*) belongs to the family Myrtaceae and is considered as most promising therapeutic fruit (13). All parts including seed, pulp, and leaves of the indigenous fruit are valuable (14). Jamun has shown its medicinal effect against many human anomalies, such as diabetes (15), cancer, inflammation, neurological issues, cardiovascular, and rheumatism (16). Jamun was assessed for its nutritional contents which showed high levels of vitamin C, anthocyanins, ellagic acid, quercetin, catechin, and caffeic acid (17). The value addition of jamun pulp has gained attention not just due to its unique flavor but also because of its promising health benefits. Therefore, many products such as cookies, noodles, jam, ready-to-serve drinks, and chapati are being produced with a wide range of flavors and enhanced nutritional quality (18).

In the present study, jamun fruit leather was used as a source for fortification. Fruit leather is a preservation method of fruits and vegetables, which is prepared by using the concentration of fruit pulp or liquids along with different ingredients. It contains pulp that is also popular among consumers because they have a high quantity of antioxidants, carbohydrates, fibers, and minerals. Moreover, fruit leather is a good approach for storing perishable fruit for a longer time (19). Regrettably, the previous literature has the efficacy studies of iron-fortified foods on anemia prevention, there may not be any studies that have specifically focussed on jamun leather fortified with iron. Additionally, the goal of the present research is to decrease post-harvest losses by converting the fruit into a valuable nutritious product that will be a chief source of iron for the anemic community of Pakistan. The current experiment is intended to evaluate the anti-anemic potential of iron-fortified jamun leather against iron deficiency anemia as well as its impact on the blood biomarkers, and liver and kidney histology in an animal model. So, the study will provide good knowledge about the use of iron-fortified jamun leather in clinical research.

## 2. Materials and methods

### 2.1. Procurement of raw material

Jamun (*S. cumini*) was collected from Ayub Agriculture Research Institute (AARI), Faisalabad, Pakistan. Sugar and pectin were obtained from the local market of Faisalabad, Pakistan, while ferrous sulfate, sodium benzoate, and citric acid were obtained from Sigma-Aldrich, St. Louis, MO, USA. The pulp of jamun fruit was extracted at the Fruits and Vegetables Laboratory, University of Agriculture, Faisalabad, Pakistan. Moreover, 40 female Sprague Dawley rats were provided by the experimental animal house of the Institute of Pharmacy, Physiology and Pharmacology, University of Agriculture, Faisalabad, Pakistan.

### 2.2. Preparation of iron-fortified jamun leather

Jamun was washed thoroughly and the pulp was prepared without seeds. Jamun pulp was homogenized with sugar (75 g/500 g pulp), pectin (15 g/500 g pulp), and citric acid (2.5 g/500 g pulp) were mixed in the sample. Then, the mixture was pasteurized at 75°C) and the iron salt was added to the mixture to yield 7.2 mg (40%) and 10.7 mg/10 g (60%) of jamun leather. The mixture was dried at 60°C with an air velocity ^@^ 3.5 m/s for 7–8 h in a cabinet dryer. The one serving of the iron-fortified jamun leather was 10 g having both 40% (7.2 mg) and 60% (10.7 mg) of Recommended Daily Allowance (RDA) of iron in women. The jamun leather was stored in labeled polythene bags until further analysis (20).

### 2.3. Experimental design

The efficacy trial was conducted to evaluate the effect of fortified jamun leather on anemia biomarkers and histology. For this purpose, 40 female Sprague Dawley rats (145–150 g) were housed in an animal room and kept for 60 days at the National Institute of Food Science and Technology, University of Agriculture, Faisalabad, Pakistan. The rats were given a normal diet for a week to acclimatize them; moreover, an environmental condition was maintained with 23 ± 2°C and 55 ± 5% relative humidity. The rats were categorized into four groups, each group having 10 rats and the grouping of rats is mentioned in Table 1. The formulated normal diet for rats was comprised of protein (10%), minerals (3%), corn oil (10%), corn starch (66%), cellulose (10%), and vitamin mixture (1%). The drug Asunra was used to induce iron deficiency anemia in rats at a dose of 10 mg/day for 14 days. All groups were fed with basal diet, but the G1 was administrated with Asunra drug throughout the study. The remaining two treatment groups G2 and G3 given basal and formulating treatment.

**Table 1:** Diet and treatment plan of animal trial.

**Groups**	**Treatments**
G_0:_ Negative control (normal rats)	Normal diet
G_1:_ Positive control (anemic rats)	Normal diet +10 mg Asunra drug
G_2_: Treatment I (anemic rats)	Normal feed + 40% iron (^*^daily value) fortified jamun leather
G_3_: Treatment II (anemic rats)	Normal feed + 60% iron (^*^daily value) fortified jamun leather

^*^RDA—Fe requirement of adult females.

### 2.4. Biochemical analyses

The rats were anesthetized in the chloroform jar and slaughtered. The blood was drawn and collected in EDTA and plain tubes for analysis. Colorimetric methods using commercial kits (Roche Diagnostics, City, Germany) were used for the measurement of serum iron level and total iron-binding capacity (TIBC). The serum transferrin levels were calculated by using the ELISA method (DRG Pharmaceuticals, GmbH, Germany). Electrochemiluminescence immunoassay (ECLIA) through Cobas e411 immunoassay analysers (Hitachi, Japan) was used to measure the ferritin levels in serum samples. The values of red cell indices such as hemoglobin (Hb), total red blood cells (TRBC), hematocrit (Hct), mean corpuscular hemoglobin (MCH), mean corpuscular volume (MCV), and mean corpuscular hemoglobin concentration (MCHC) were calibrated by using an automated Blood analyser (21).

### 2.5. Histopathology evaluation

For this purpose, the rats were dissected, kidney and liver were cut into smaller blocks and stored in formalin for 3 days. The tissues were trimmed with 0.3–0.5 cm wide 3.13.3 and dehydrated. The dehydrated samples were immersed in chloroform solution overnight and infiltrated with histological wax molds. After solidification excess of the paraffin was trimmed using a razor blade. The slides were placed in xylene for 3–4 min and afterwards dipped in Erlich's Hematoxylin stain for 30 min to provide a contrast to the tissue sections, the purpose was to make tissues visible for the microscopic evaluation. Afterwards, the coverslip was mounted over the tissue on the slides to make them ready for the examination (22).

### 2.6. Statistical analysis

All the data for the above-mentioned parameters were taken in triplicate. The statistical analysis for parameters measured and the blood biochemistry before and after treatment was analyzed using a TWO-WAY ANOVA test followed by an LSD *post-hoc* test with a significance value of *P* < 0.005. All the obtained results are expressed as mean ± SD.

## 3. Results

### 3.1. Fortification effect on blood biochemistry

**Figure 1:**
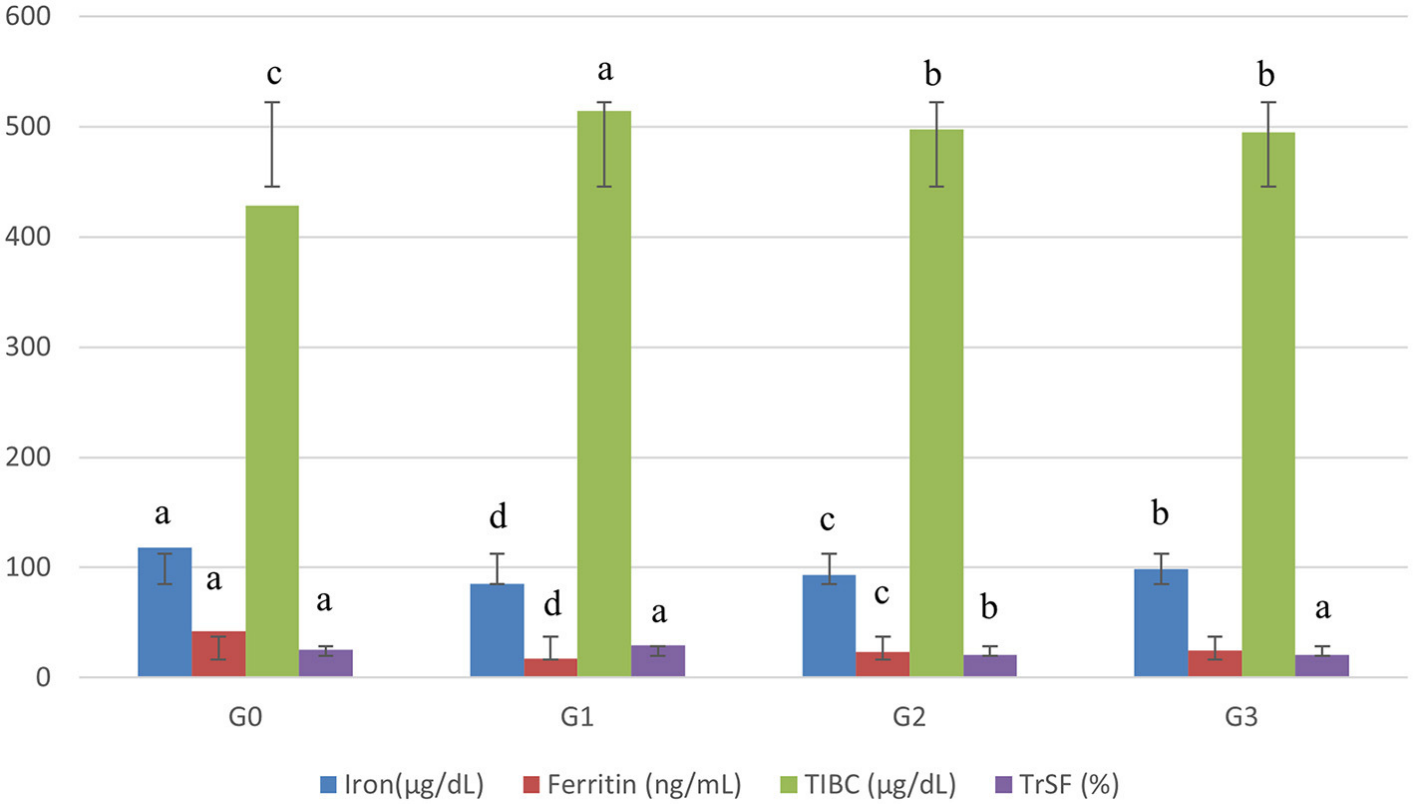
Effect of treatments on blood biochemistry of rats. G_0_, Negative control = Normal feed; G_1_, Positive control = Normal diet + Asunra drug; G_2_, anemic rats + 40 mg iron/kg of feed; G_3_, anemic rats + 60 mg iron/kg of feed. The small letters designate significant comparison between study groups.

The data for the serum iron (SI) of female Sprague Dawley rats after 60 days trial are depicted in Figure 1. The statistical analysis (*P* < 0.05) of SI in the Asunra-induced anemic rat group depicted significant differences among the treated group for 60 days study. These showed that drug administration in the normal diet had a negative impact by chelating the body's naturally occurring iron, leading to iron deficiency anemia. However, the administration of treatments along with a normal diet increased serum iron in G_2_ (9.23%) and G_3_ (15.78%), respectively. The serum iron recorded for G_3_ (98.68 ± 2.88 μg/dL), which was significantly higher than G_2_ (93.10 ± 2.72 μg/dL).

The mean relating to serum ferritin expounded significant differences (*P* < 0.05) for anemic rats' groups. The serum ferritin levels were more restored in G_3_ (24.54 ± 1.98 ng/mL) than in G_2_ (23.39 ± 2.05 ng/mL). Moreover, the data elucidated that the levels of serum ferritin of G_2_ and G_3_ were improved from 41 to 44% and 32 to 39% when compared with G_0_ and G_1_, respectively.

Remarkable differences (*P* < 0.05) were recorded related to total iron-binding capacity (TIBC) among groups. The lowest level of TIBC in anemic rats was noted in G_3_ (495.15 ± 8.25 μg/dL), followed by G_2_ (497.59 ± 8.34 μg/dL). TIBC levels in G_2_ and G_3_ were increased by 3.3–3.7 and 13–14% when compared with the anemic rat group (G_1_) and normal rat group (G_0_) groups (Figure 1).

The results of serum transferrin (TrSF) are given in Figure 1. Significant differences (*P* < 0.05) were noticed among all groups. From the results, it was observed that the rats depending on the iron-fortified jamun leather G_2_ (20.39 ± 2.17), and G_3_ (20.63 ± 2.21) had a lower level of serum transferrin as compared to G_1_ (29.61 ± 2.21).

### 3.2. Impact of iron-fortified jamun leather on red cell indices

The red cell indices including hemoglobin (Hb), hematocrit (Hct), total red blood count (TRBC), mean corpuscular hemoglobin concentration (MCHC), and mean corpuscular volume (MCV) is shown in Table 2. The statistical analysis showed that the treatments had a significant effect on the red cell indices of experimental groups. The hemoglobin concentration was decreased (27%) in G_1_ as compared to G_0_ and increased in G_2_ (18%) and G_3_ (20%) in comparison to positive control G_1_. The restoration of Hb concentration from anemia was more pronounced in G_3_.

**Table 2:** Impact of iron-fortified jamun leather on red cell indices of rats.

**Experimental groups**	**Hb (g/dL)**	**Hct (%)**	**TRBC (M/μL)**	**MCV (femtometer/cell)**	**MCHC**
G_0_	13.69 ± 0.26^a^	42.87 ± 1.44^a^	7.40 ± 0.19^a^	55.52 ± 1.11^a^	32.31 ± 1.21^a^
G_1_	9.89 ± 0.39^d^	37.65 ± 1.35^d^	6.94 ± 0.18^c^	51.89 ± 1.12^c^	28.07 ± 1.20^c^
G_2_	12.08 ± 0.26^c^	38.54 ± 1.21^c^	7.20 ± 0.14^b^	53.38 ± 1.13^b^	30.67 ± 1.15^b^
G_3_	12.41 ± 0.32^b^	39.30 ± 1.66^b^	7.16 ± 0.17^b^	53.52 ± 1.13^b^	30.65 ± 1.21^b^

G_0_, Negative control = Normal feed; G_1_, Positive control = Normal diet + Asunra drug; G_2_, anemic rats + 40 mg iron/kg of feed; G_3_, anemic rats + 60 mg iron/kg of feed. The small letters designate significant comparison between study groups.

Total red blood count and hematocrit were decreased (6 and 12%) in positive control G_1_ when compared to G_0_. The treatments helped in improving TRBC concentrations in G_2_ and G_3_ by 4.38 and 2.36%. Similarly, hematocrit levels were increased by (2.36%) G_2_ and (3.17%) G_3_ when compared to positive control G_1_. The treatments fed with iron-fortified products achieved an improvement in the MCV and MCHC levels. The data showed MCV levels were 2.87 and 3.14% higher in G_2_ and G_3_ than in G_1_. Similarly, the mean corpuscular hemoglobin concentration (MCHC) levels were increased in G_2_ at 9.26 and G_3_ at 9.19 as compared to the positive control group.

### 3.3. Hepatic and renal histopathological evaluation

**Figure 2:**
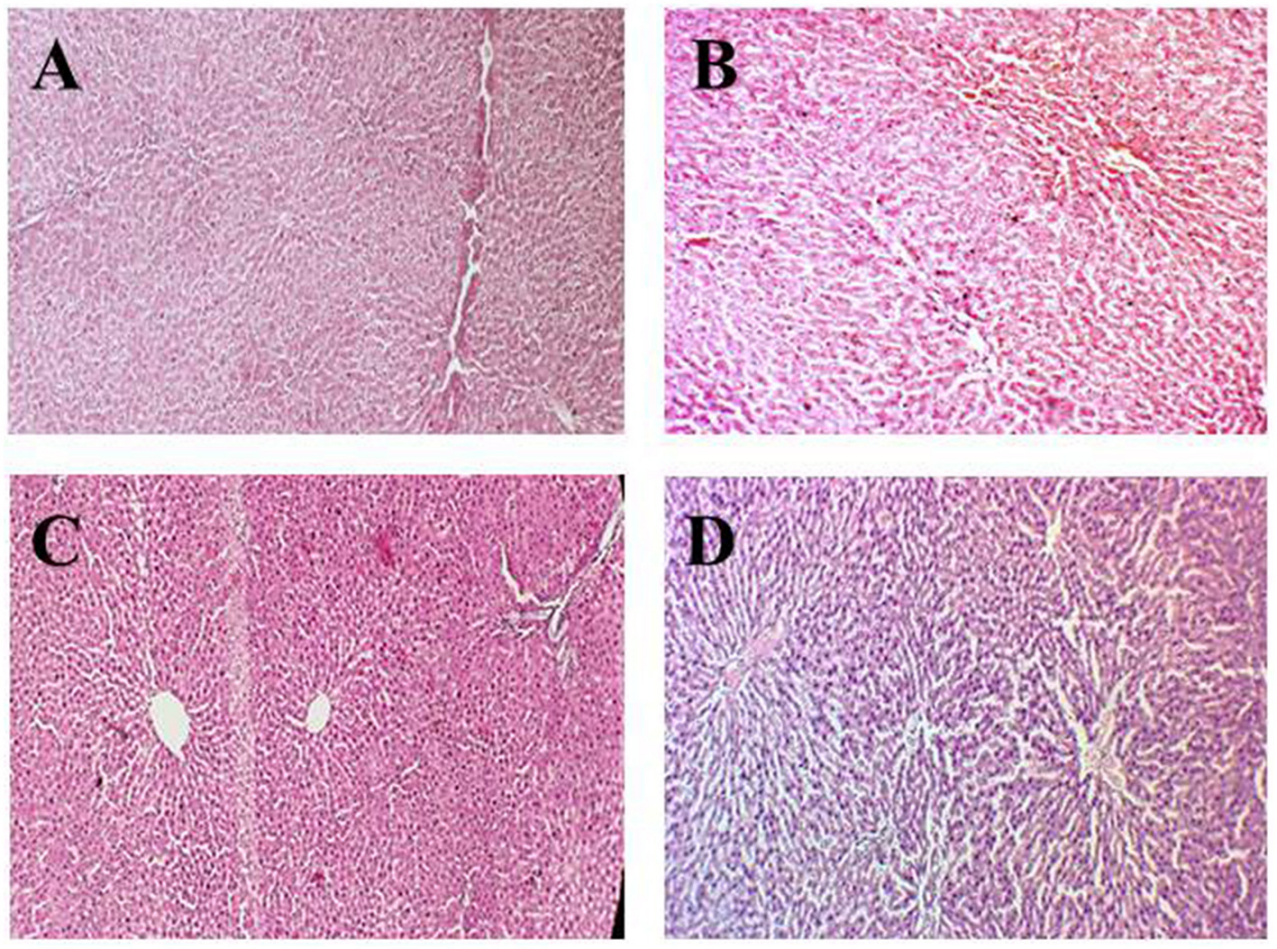
Histopathological indications of the renal parenchyma. **(A)** (G_0_) Negative control, **(B)** (G_1_) Positive control, **(C)** (G_2_) Anemic rats + 40 mg iron/kg of feed, **(D)** (G_3_) Anemic rats + 60 mg iron/kg of feed.

Figures 2A–D presents the histopathology of the rat kidney in the experimental groups. Figure 2A shows the negative control group (G_0_) kidney tissue with a normal appearance of the renal parenchyma, normal cortex, and glomerulus. Moreover, the renal tissue had tubules with well-preserved architecture lined by cuboidal epithelial cells. Figure 2B represents renal tissues of anemic rats with necrotic changes at the cellular level, a coagulative necrosis which is the denaturation of proteins present in the kidney was observed in microscopic examination. Moreover, the normal color of the cytoplasm faded to light pink color, and overall cellular architecture was compromised.

In Figure 2C, the renal section of rats treated with 40% fortified jamun leather (G_2_) shows normal kidney architecture and well-preserved kidney tubules. But at some points in tissues as well as kidney tubules, some necrotic foci had been observed due to the induction of iron deficiency anemia using the drug. In Figure 2D, the kidney section of rats treated with 60% fortified jamun leather (G_3_) shows improved and restored architecture with intact cell boundaries, and no definite necrosis was observed.

**Figure 3:**
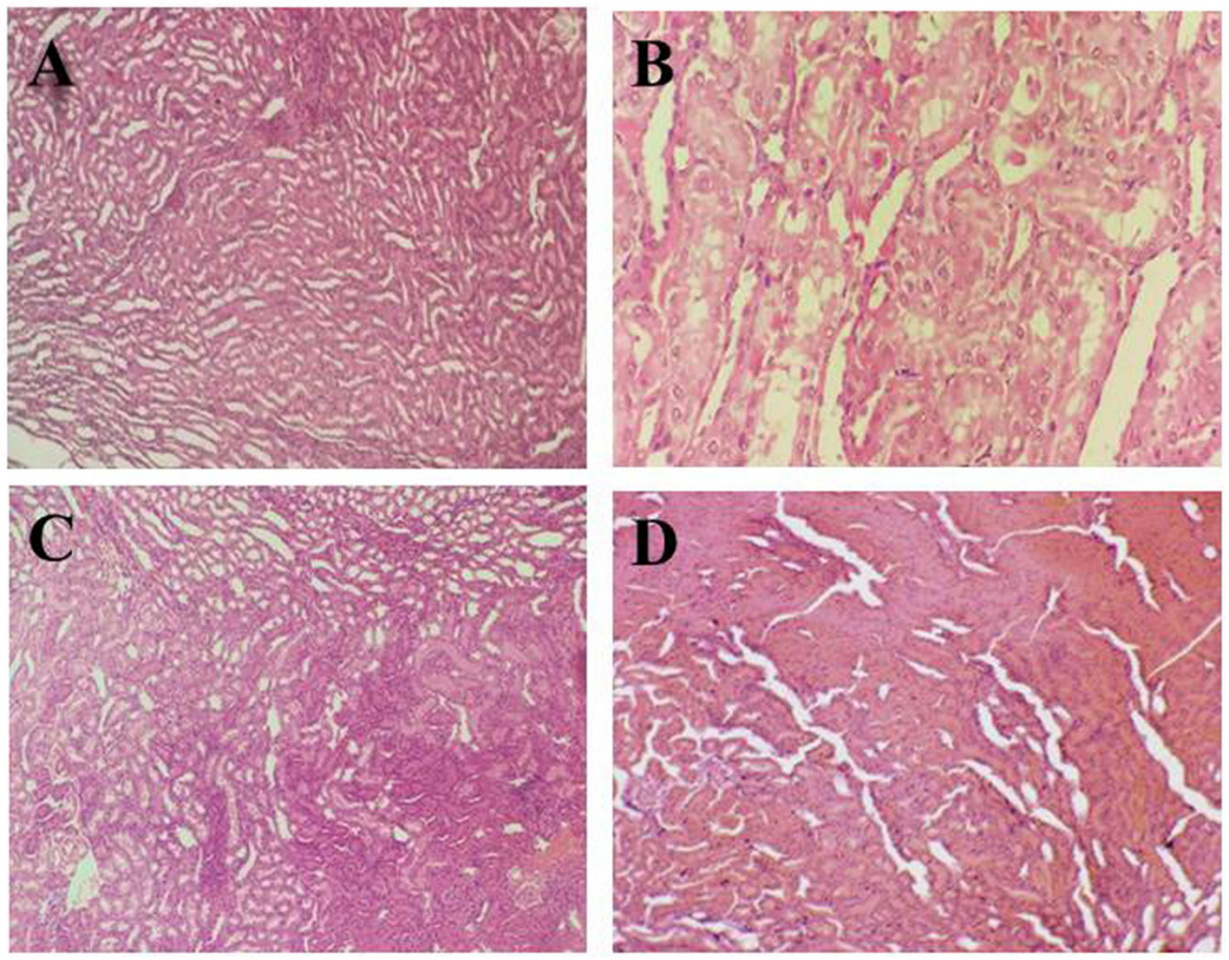
Histopathological indications of liver tissue. **(A)** (G_0_) Negative control, **(B)** (G_1_) Positive control, **(C)** (G_2_) Anemic rats + 40 mg iron/kg of feed, **(D)** (G_3_) Anemic rats + 60 mg iron/kg of feed.

Figures 3A–D illustrates the hepatic histopathology of rats in different groups. The tissue image of the negative control group (G_0_) in Figure 3A shows the normal hepatic architecture, hepatocytes in the tubular pattern, and central vein. Overall, an intact parenchyma structure was observed. The photomicrograph (Figure 3B) of positive control (G_1_) shows inflamed neutrophils in tissue lymphocytes along with balloon-like degradation of damaged hepatocytes leading to necrosis of liver cells in hepatotoxic rats.

In comparison to the anemic group (G1), Figure 3C of the rat group (G2) demonstrates well-preserved cell structure and balloon degradation. Polyhedral hepatocytes and dark color lymphocytes were observed which indicated the recovery toward a healthy liver. In Figure 3D, the liver section of group (G_3_) shows disarranged hepatic sinusoids, hepatocytes having distinct cell borders, and balloon degradation. The reason behind the changes in liver tissue structure may be due to the drug's effect on the rats and slow recovery from anemia.

## 4. Discussion

Women face more iron deficiency and tend to have anemia at any age of life. Iron supplementation has been considered the most common practice to alleviate iron deficiency, but food fortification is a more practical and effective strategy to combat deficiency at a large scale (23). The effects of prepared iron-fortified jamun leather treatments were investigated in the present study using a female Sprague Dawley rat model with iron-deficient anemia. A study was conducted to determine the effect of 2 mg/kg ferrous sulfate supplementation in anemic rats and measured the blood biomarkers including total iron-binding capacity, serum iron, transferrin, and ferritin levels. The researchers noticed that ferrous sulfate increased serum transferrin and replenished the iron level (24).

Transferrin is the protein of the body responsible for the binding of iron and its transport. The low value of transferrin is an indicator of iron deficiency anemia and vice versa is the overload of iron or hemochromatosis (25). Total iron-binding capacity (TIBC) is the biomarker for the detection of iron deficiency anemia. The test indicates the capacity of body protein transferrin to carry iron. Moreover, the greater the value of TIBC, the lower the level of iron in the body causing iron deficiency anemia (26). The results of our study are quite in line with the previous literature. He et al. (27) have investigated that anemic rats fed on 4 mg/kg ferrous sulfate supplementation can easily restore the total iron-binding capacity and transferrin concentration. They compared transferrin saturation in the blood of anemic rats as 3.37%, the ferrous sulfate fed group at 37.90%, and total iron-binding capacity in the anemic was 113.91 μmol/L, ferrous sulfate fed group at 72.91 μmol/L.

Serum ferritin is considered an indirect evaluator of the iron reserves in the body as the depletion might be the indicator for anemia (28). Elzamzamy and Mostafa studied iron bioavailability in iron-deficient rats using different fruit and vegetable juices supplemented with ferrous sulfate. They observed a remarkable increase in the level of serum iron, ferritin, hemoglobin, and hematocrit (29). Another research carried out by Asperti et al. prepared a formulation having phospholipids containing ferric pyrophosphate sucrosomial and ferrous sulfate supplementation in normal and iron-deficient anemic rats. They reported an improvement in the levels of iron in the group treated with ferrous sulfate as well as in the group treated with sucrosomial i.e., 100–250 μg/dL. They concluded that both supplementations proved to be effective in the replenishment of iron in anemic rats (30). The results of the present research are similar to Alferez et al. (31) have proven that a dose of 45 mg Fe/kg diet dietary iron improves the anemia biomarkers. They mentioned improvement in transferrin (29.5–40.3%) hemoglobin concentrations (116–123 g/L) and hematocrit (46.3–50%). Moreover, the MCV, MCHC, and total red blood count levels were increased due to the administration of iron in the feed.

We have identified that fortified jamun leather helped in increasing serum iron, hemoglobin, transferrin, and TIBC of anemic rats. Similarly, a group of scientists inoculated iron nanoparticles in food to reverse the levels of iron-depleted during iron deficiency anemia. It has been found that the iron nanoparticles helped in the restoration of the concentrations of transferrin, serum iron, and ferritin (32). In our study, the transferrin levels were almost the same in G_a_ and G_3_ after administration of fortified jamun leather even though the fortificant concentration was significantly different. Kyyaly et al. also observed slight differences in the transferrin concentration when fed with high levels of iron (33).

The supplementation of ferrous sulfate salt improved the levels of hemoglobin in Wistar rats thus helping to manage iron deficiency anemia (1). Cable et al. noticed an improvement in the transferrin levels in anemic women. A steady decline in serum transferrin levels indicates the anemia is being cured and a lesser amount of iron is needed by the body (34). In Indonesia, iron deficiency anemia is very common among women and children. Thus, ingestion of iron-fortified milk and noodles by children of age 6–59 months decreased iron deficiency anemia (35). Iron is associated with gut health; deficiency of iron may lead to dysfunctional metabolism and cognition. Ingestion of iron-fortified tempeh markedly raised the hemoglobin and serum iron concentrations as well as *cecal lactobacillus* count was improved (36). The researchers conducted a study on the effect of iron deficiency anemia on lipid peroxidation and DNA stability and recorded an increase in hemoglobin concentration, red blood cells level, hematocrit, and mean corpuscular volume but without affecting the DNA stability and inducing oxidative stress (37). The long-term administration of iron-fortified food products did not cause any oxidative damage to DNA and liver protein. Moreover, the fortification of iron helped in the maintenance of body regulatory antioxidants affected by iron deficiency anemia (38).

Under hypoxic conditions, the body is more reactive to free oxygen species, and this imbalance in the body leads to oxidative stress in the body. Oxidative stress is closely linked with the pathophysiology of iron deficiency anemia. Oxidative stress alters function and disrupts organ structure (39). Previous researchers highlighted the non-toxic effect of iron fortification on renal and hepatic tissues of normal and anemic rats (1, 40). Fortification of biscuits can be an affordable strategy to combat prevailing deficiencies. The intake of iron-fortified biscuits restored the serum iron, hemoglobin, and red blood cells in anemia. The microscopic biopsy of hepatorenal tissues had no significant effect on the fortificant (41). The histopathological studies presented are closely related to the research of Mohammed et al. (42), who observed alterations in the liver of anemic rats such as the dilation of veins, hemorrhage in the central vein, loss of hepatocytes structure, and the aggregation of lymphocytes. While in the treated group, hepatocytes appeared to be close to the normal hepatocytes and intact hepatic sinusoids. The researchers predicted that there was a recovery in the tissue structure of the liver of iron supplemented group. Similarly, intake of iron-rich drinking water preserved the normal structure of liver cells, central vein, and hepatocytes as compared to the liver of the anemic group having prominent hepatocellular necrosis along with neutrophil infiltration and sinusoidal congestion. Moreover, in the case of renal histopathology, they observed iron deposition in neither the anemic nor treated group (43). Adding iron-fortified liposomes to the daily diet of experimental rats' significantly replenished hemoglobin concentrations with no histopathological toxicity indication (44).

In conclusion, our histopathological analyses of kidney and liver tissues had no changes in architecture. These results agree with the findings of Darwaish et al. (45), who discussed the efficiency and safety of nutrient delivery to the body. However, it is crucial to have information and verify the fortificant safety before use in human clinical trials. Based on the findings of serum analysis and histopathological examination, it is concluded that iron-fortified jamun leather can effectively mitigate iron deficiency anemia.

## 5. Conclusion

In summary, we conclusively established that the iron-fortified jamun leather improves serum biomarkers such as serum iron, ferritin, hemoglobin, and total red blood cells. Furthermore, it normalizes serum transferrin and total iron-binding capacity levels. Also, the microscopic study of kidney and liver tissue revealed that the iron-fortified jamun leather did not impart any damaging effect on the cell structure. These findings confirmed that iron-fortified jamun leather could be used as an effective approach against iron deficiency anemia. Further research in this area may contribute to combating anemia and improving the overall health and wellbeing of populations vulnerable to iron deficiency.

## Data availability statement

The original contributions presented in the study are included in the article/supplementary material, further inquiries can be directed to the corresponding authors.

## Ethics statement

The animal study was reviewed and approved by Pakistan Biosafety Committee 2005, Punjab Biosafety Rules 2014, and Bioethics Protocols, Office of Research, Innovation and Commercialization, University of Agriculture, Faisalabad, Pakistan.

## Author contributions

NN: conceptualization, data curation, formal analysis, resources, and writing—original draft. MK: supervision, conceptualization, and writing—review and editing. MS: writing—review and editing and software. MF: writing—review and editing. All authors contributed to the article and approved the submitted version.
